# Healthcare provider perspectives on integrating peer support in non-dialysis-dependent chronic kidney disease care: a mixed methods study

**DOI:** 10.1186/s12882-022-02776-w

**Published:** 2022-04-18

**Authors:** Shannan Love, Tyrone G. Harrison, Danielle E. Fox, Maoliosa Donald, Nancy Verdin, Brenda R. Hemmelgarn, Meghan J. Elliott

**Affiliations:** 1 Department of Medicine, Cumming School of Medicine, University of Calgary, TRW Building, 3280 Hospital Drive NW, Calgary, Alberta T2N 4Z6 Canada; 2 Department of Community Health Sciences, University of Calgary, Calgary, Alberta Canada; 3 Medicine Strategic Clinical Network, Patient & Family Advisory Council, Alberta Health Services, Calgary, Alberta Canada; 4 Patient and Community Engagement Research (PaCER) Program, O’Brien Institute for Public Health, University of Calgary, Calgary, Alberta Canada; 5 Department of Medicine, University of Alberta, Edmonton, Alberta Canada

**Keywords:** Chronic kidney disease, Peer support, Mixed methods

## Abstract

**Background:**

Peer support complements traditional models of chronic kidney disease (CKD) care through sharing of peer experiences, pragmatic advice, and resources to enhance chronic kidney disease self-management and decision-making. As peer support is variably offered and integrated into multi-disciplinary CKD care, we aimed to characterize healthcare providers’ experiences and views on peer support provision for people with non-dialysis-dependent CKD within Canada.

**Methods:**

In this concurrent mixed methods study, we used a self-administered online survey to collect information from multi-disciplinary CKD clinic providers (e.g., nurses, nephrologists, allied health professionals) on peer support awareness, program characteristics and processes, perceived value, and barriers and facilitators to offering peer support in CKD clinics. Results were analyzed descriptively. We undertook semi-structured interviews with a sample of survey respondents to elaborate on perspectives about peer support in CKD care, which we analyzed using inductive, content analysis.

**Results:**

We surveyed 113 providers from 49 clinics. Two thirds (66%) were aware of formal peer support programs, of whom 19% offered in-house peer support through their clinic. Peer support awareness differed by role and region, and most referrals were made by social workers. Likert scale responses suggested a high perceived need of peer support for people with CKD. Top cited barriers to offering peer support included lack of peer support access and workload demands, while facilitators included systematic clinic processes for peer support integration and alignment with external programs. Across 18 interviews, we identified themes related to peer support awareness, logistics, and accessibility and highlighted a need for integrated support pathways.

**Conclusions:**

Our findings suggest variability in awareness and availability of peer support among Canadian multi-disciplinary CKD clinics. An understanding of the factors influencing peer support delivery will inform strategies to optimize its uptake for people with advanced CKD.

**Supplementary Information:**

The online version contains supplementary material available at 10.1186/s12882-022-02776-w.

## Background

Chronic kidney disease (CKD) is a complex condition that affects 13% of the population globally and, in its most advanced stage, can result in kidney failure requiring dialysis or transplantation as life-sustaining therapy [1]. The varied and dynamic nature of CKD presents unique challenges to living with this condition and highlights a need for tailored management approaches to meet individuals’ support needs and slow disease progression [2, 3]. Peer support for persons living with CKD has been identified as one potential strategy for assisting with kidney disease self-management by supporting their informational needs, emotional well-being, and self-efficacy [3–5].

Peer support may augment traditional models of multi-disciplinary CKD care by enabling peer validation of patients’ experiences and exchange of pragmatic resources for living well with their disease [4–8]. Peer support can further influence decision-making through offering practical advice, sharing experiences, and normalizing kidney replacement therapies for people with kidney failure [9–12]. Delivery of peer support can take place as informal supportive encounters between peers [13], or alternatively through formalized programs within kidney care programs or in partnership with external organizations [14, 15]. Despite its benefits, the role of peer support in the comprehensive care of persons with non-dialysis-dependent CKD remains unestablished [16]. Opportunities for informal peer connection in non-dialysis-dependent CKD are uncommon, and uptake of formal peer support programs has traditionally been low with variability in how it is promoted and offered [15–17].

Limited research on peer support for people with kidney disease suggests that variability in awareness and promotion within kidney care programs poses a barrier to peer support uptake [14]. While a national, telephone-based peer support program offered by the Kidney Foundation of Canada (i.e., ‘Kidney Connect’) has been the main source of kidney-focused peer support for nearly 10 years in Canada, little is known about how multi-disciplinary CKD clinics promote uptake of this program and integrate other models of peer support into patient care [18, 19]. For people with advanced, non-dialysis-dependent CKD, the longitudinal surveillance and established relationships within multi-disciplinary CKD clinics make this an appealing setting for enhancing peer support awareness and concerted approaches to its delivery [19–22]. Given the lack of uniform approach to offering, promoting, and integrating peer support within multi-disciplinary CKD clinics, this study aimed to characterize providers’ experiences and views related to the provision of peer support for people with non-dialysis-dependent CKD within the Canadian context.

## Methods

### Study design

We used a concurrent mixed methods approach to understand how peer support is perceived and delivered in multi-disciplinary CKD clinics across Canada [23]. This involved use of survey methodology for quantitative data collection alongside a qualitative descriptive approach through individual interviews. We triangulated and integrated findings in the interpretation phase to elaborate on complementary concepts and provide depth of insight. We followed the Mixed Methods Article Reporting Standards (MMARS) and the Consolidated Criteria for Reporting Qualitative Research (COREQ) for reporting results (Additional file 1) [24, 25]. Ethics approval was granted by the University of Calgary Conjoint Health Research Ethics Board (REB20-1159).

### Sample and recruitment

Eligible participants included healthcare providers (e.g., nurses, nephrologists, allied health professionals) from multi-disciplinary CKD clinics across Canada. We purposively sampled eligible providers through the CKD Clinic Network, a pan-Canadian organization of providers and managers from multi-disciplinary CKD clinics who care for persons with advanced, non-dialysis-dependent CKD [19, 26]. A network coordinator distributed the study to individuals from 66 CKD clinics by email. Interested members were asked to complete the online survey and forward the invitation to colleagues in their clinics and other contacts within their discipline (i.e., snowball sampling). The survey was administered between October and December 2020. Interviews were completed with survey participants who indicated their interest to expand on ideas around peer support in CKD care.

### Data collection

#### Online survey

A self-administered online survey was developed by the investigators based on findings from previous work and related peer support and self-management literature (Additional file 2) [27]. The survey was generated and offered in English using the Qualtrics™ survey platform (Qualtrics, Provo, UT) and contained 40 questions that took approximately 10 min to complete. Most questions were not compulsory, and as such, response rates varied for each question. Questions were grouped into the following categories as they relate to peer support delivery: (i) awareness and availability; (ii) characteristics of in-house programs, if available; (iii) processes for referral; (iv) barriers and facilitators; and (v) perceived value.

The survey was assessed for content and face validity through iterations of revisions and pilot testing with two CKD clinic providers and one clinician researcher. Most questions required selection from pre-determined options but offered open-ended text boxes to elaborate on responses if desired. Participants selected barriers and facilitators from a pre-determined list generated from literature review by checking those perceived to apply, then ranking their selections from highest to lowest [6, 7]. Respondents indicated perceived need, interest, and value of peer support through a Likert scale ranging from 0 (low) to 10 (high). Consent to participate was outlined on the survey instruction page and implied by advancing through survey questions.

#### Semi-structured interview

Interested survey respondents who provided verbal consent completed semi-structured interviews by telephone or virtual Microsoft Teams™ platform, as participants resided across Canada and COVID-19 precautions were in place. A research team member (SL) experienced in qualitative research and with no prior relationships with participants completed all interviews, which were audio-recorded and lasted approximately 30-40 min. A question guide was used to prompt discussion about the promotion and integration of peer support programs in CKD clinic care and their perceived need to address identified care gaps (Additional file 3). Interview recruitment ceased once all available participants had been interviewed and data saturation had been attained. We determined data saturation as the point at which a breadth of participants (i.e., varied demographic characteristics) and perspectives related to peer support delivery had been captured, and little or no new information emerged during analysis of interview data [28].

### Data analysis

#### Quantitative

We summarized participant and CKD clinic characteristics with counts and proportions for categorical and dichotomous variables. We examined the proportion of respondents who were aware of peer support and those whose clinic offered peer support internally. Exploratory analyses examined whether there were differences in response based on respondent role, region, or years of clinical experience. For these comparisons, we used Pearson chi-square of Fishers exact statistical tests (depending on minimum category frequency), with a *p*-value for significance of 0.05. Individual rankings for the 8 listed barriers and facilitators were determined through a process of reverse scoring, such that items ranked first received a score of 8, those ranked last received a score of 1, and those not ranked received a score of 0. The aggregate priority score was calculated for each barrier and facilitator, which were then ranked by overall priority. We examined the distribution of our Likert survey questions with violin plots, which included response kernel density visualization in addition to median and interquartile range of responses. For all statistical analyses, we used Stata software v16 and 17 (StataCorp) [29].

#### Qualitative

We used conventional content analysis involving systematic, line-by-line coding and theme characterization to provide a descriptive account of participants’ views [30]. Analysis was inductive in that codes and themes were derived directly from the data. Interview transcripts and open-ended survey questions were uploaded to NVivo 12 (QSR International Pty Ltd) to facilitate data organization [31]. Initial transcripts were coded in duplicate by research team members (SL, MJE). A preliminary coding framework was generated and applied to subsequent transcripts, with revisions made through team discussions. Discrepancies in coding were resolved through investigator discussion and consensus. We then generated preliminary themes which were checked against the dataset. Frequency counts from relevant codes were used to calculate the top reported barriers and facilitators.

## Results

### Descriptive survey results

#### Survey demographic and clinic characteristics

A total of 113 providers from 49 unique clinics across 10 Canadian provinces/territories completed the online survey (Table 1). Most respondents provided care to patients with non-dialysis-dependent CKD (84%), with many also providing care to individuals with other categories of kidney disease. Almost half of respondents were nurses (48%), with CKD clinics having a varied multi-disciplinary team composition.

**Table 1:** Characteristics of participants (survey and interviews)

Characteristic	Survey (*n* = 113) Number (%)	Interviews (*n* = 18) Number (%)
Respondent role in CKD clinic		
Nurse	54 (47.8)	5 (27.8)
Social worker	17 (15.0)	8 (44.4)
Nephrologist	13 (11.5)	2 (11.1)
Manager	10 (8.8)	3 (16.7)
Dietitian	9 (8.0)	
Support staff	4 (3.5)	
Pharmacist	3 (2.7)	
Other (i.e., Indigenous navigator)	1 (0.9)	
Respondent length of time in current position		
Less than 1 year	15 (13.3)	
1-5 years	38 (33.6)	
6-10 years	28 (24.8)	
More than 10 years	32 (28.3)	
Geographical region where clinic located		
British Columbia	50 (44.2)	5 (27.8)
Alberta, Saskatchewan, Manitoba	30 (26.6)	6 (33.3)
Ontario, Quebec	20 (17.7)	5 (27.8)
Atlantic Canada	9 (8.0)	2 (11.1)
Territories	2 (1.8)	
City size where clinic located		
Less than 100,000	35 (31)	
100,000-499,999	35 (31)	
500,000-1,000,000	8 (7.1)	
More than 1,000,000	34 (30.1)	
Type of CKD patients seen in clinic^a^		
Primarily non-dialysis CKD:		
G1-G3b	74 (65.5)	
G4 and G5 Non-dialysis	94 (83.2)	
Other categories of kidney disease:		
G5D Hemodialysis	41 (36.3)	
G5D Peritoneal dialysis	32 (28.3)	
G5T Transplant	21 (18.6)	
Other (e.g., kidney stones, AKI, transplant donors)	6 (5.3)	
Unsure	1 (0.9)	
CKD clinic care team members^b^		
Registered nurse	111 (98.2)	
Nephrologist	107 (94.7)	
Dietitian	106 (93.8)	
Social worker	101 (89.4)	
Support staff (e.g., Unit clerks, Renal technicians)	67 (59.3)	
Pharmacist	65 (57.5)	
Licensed practical nurse, Nursing assistant	32 (28.3)	
Nurse practitioner	17 (15.0)	
Kinesiologist	3 (2.7)	
Occupational therapist	2 (1.8)	
Other (e.g., Indigenous navigator, Psychologist)	7 (6.2)	

Missing data exists in some fields. All proportions are reported with denominator *n* = 113

*Abbreviations*: *AKI* acute kidney injury, *CKD* chronic kidney disease

^a^Many respondents provided care to patients at other stages of kidney disease in addition to non-dialysis CKD, therefore chose more than one response. ^b^Many respondents chose more than one response to represent the composition of the multi-disciplinary team within their CKD clinic

#### Peer support program characteristics

Seventy-five of the 113 (66%) respondents reported being aware of formal peer support programs for patients with non-dialysis-dependent CKD, the most common being the Kidney Foundation of Canada’s ‘Kidney Connect’ program (81%). Nearly half (43%) were aware of informal peer support encounters through their clinic (e.g., during educational sessions). Of the 75 respondents aware of formal peer support programs, 59 (79%) indicated they had referred clinic patients to one, and 14 (19%) reported offering in-house peer support through their clinic. Most in-house programs targeted individuals with CKD and were facilitated by patient volunteers, providers, partner organizations, or a combination. Most in-person peer support programs offered within CKD clinics had been suspended due to the COVID-19 pandemic, with limited ability to switch to alternate, virtual formats (Additional file 4).

#### Peer support processes

**Fig. 1:**
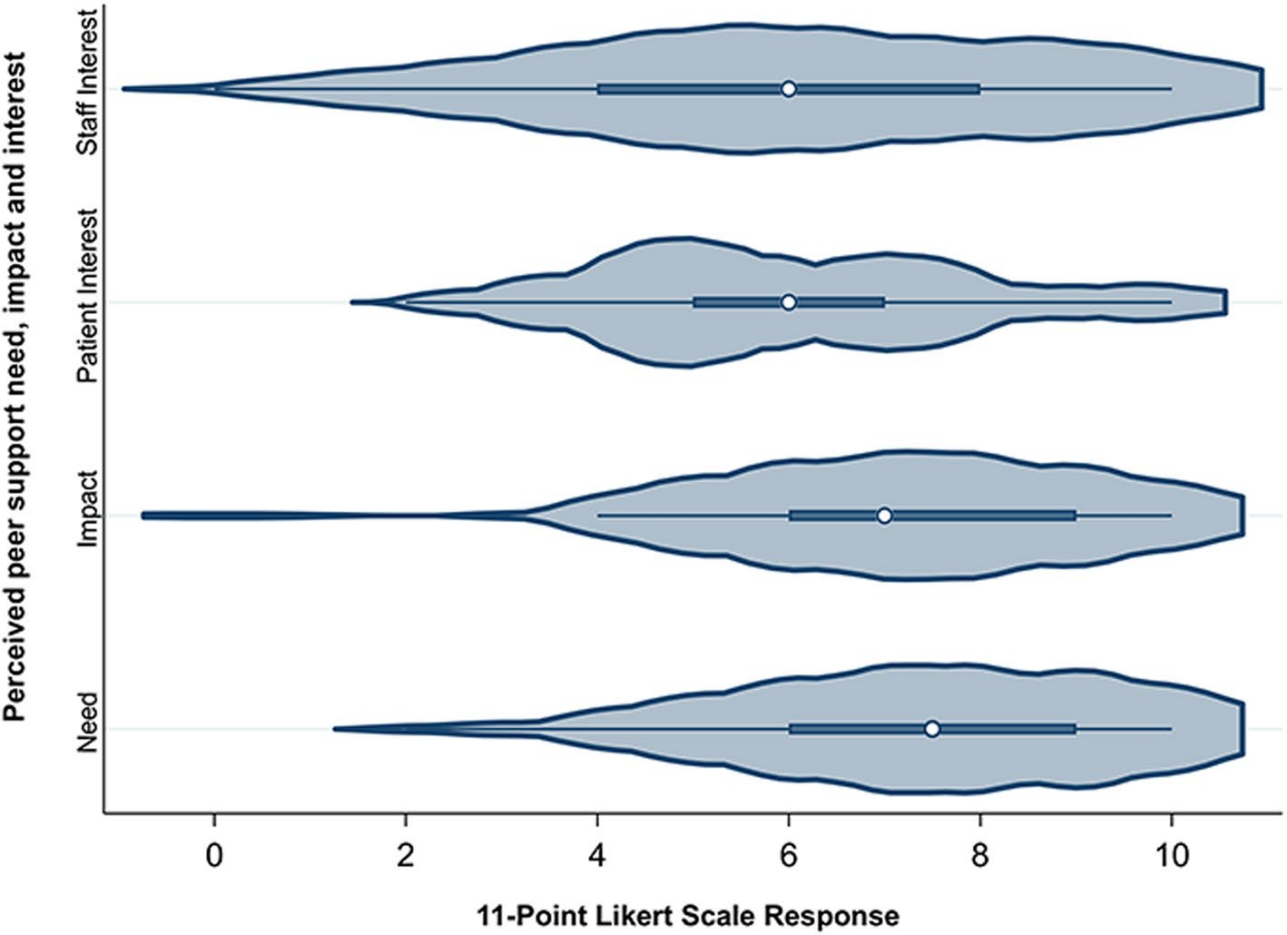
Responses to perceived need, staff interest, patient interest, and impact questions on 11-point Likert scales. The Likert scales ranged from 0 = no need/interest/impact to 10 = high need/interest/impact. In each violin plot, the white point estimate represents the median, flanked by the interquartile range represented by the thicker line. The kernel density of responses is also visualized with the margins of each plot

Approximately half of respondents learned about peer support programs informally from other team members (52%) or from promotional material provided by external organizations (53%). Patients and caregivers learned about peer support opportunities during clinic through staff member prompting (83%), brochures (59%), or posters in waiting rooms (40%). All three domains of peer support – informational, emotional, and appraisal [32] - were reported equally as factors that would prompt peer support discussions at various times, including during clinic intake then periodically thereafter (28%), situationally when patients required additional support (51%), or when patients were faced with significant decisions (36%). Although staff introduced peer support during care encounters, patients often had to initiate peer support referrals themselves (65%). Peer volunteers were rarely involved in introducing or referring patients to peer support (Additional file 5). Likert scale responses consistently suggested high perceived patient need and staff interest in peer support, with slightly lower rated patient interest and impact of peer support (Fig. 1 and Additional file 6).

**Fig. 2:**
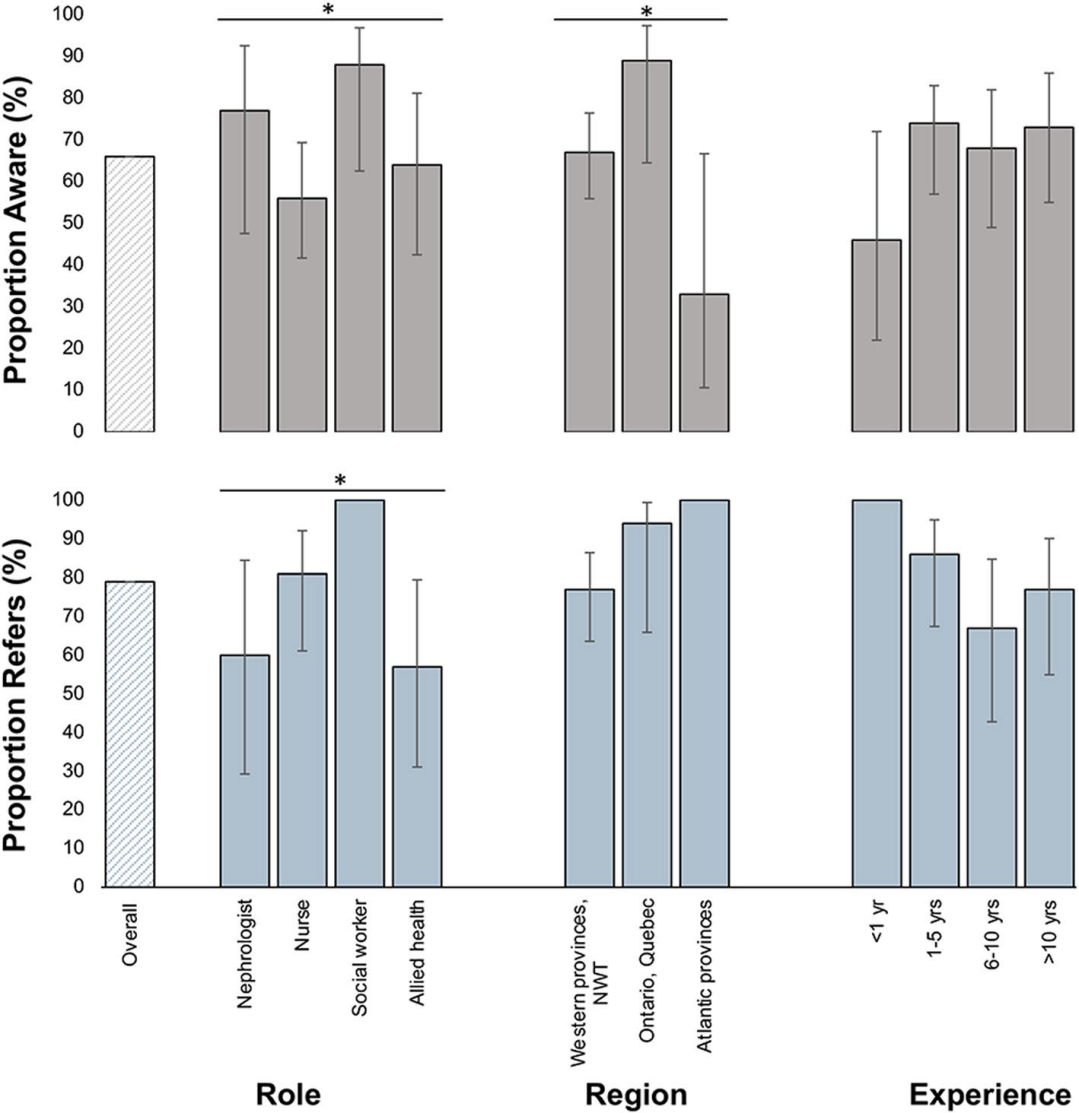
CKD peer support awareness and referral patterns stratified by role, location, and years of experience. The proportion of healthcare providers aware for each of these categories is based on the denominator of 113 respondents. The proportion of healthcare providers who refer to peer support is based on the denominator of 75 respondents. Abbreviation: NWT, Northwest Territories. * denotes significance *p* < 0.05

We identified significant differences in respondents’ awareness of peer support by role and region in Canada but not by years of experience (Fig. 2). Most social workers and nephrologists were aware, with approximately half of nurses and two thirds of other allied health professionals reporting awareness (*p* = 0.03 for differences in awareness by role). Peer support awareness was highest in Ontario and Quebec (89%) as compared to Western (68%) and Atlantic (33%) provinces. When examining the same factors influencing referral to peer support programs, we found significant differences by participant role but not region or years of practice. Of those aware of peer support programs, social workers and nurses most frequently referred patients to peer support, whereas referrals from nephrologists and other allied health professionals were less common (*p* = 0.008 for differences in referrals by role) (Fig. 2).

### Qualitative findings

Among survey respondents, we conducted semi-structured interviews with 8 social workers, 5 nurses, 3 managers, and 2 nephrologists (Table 1). While interview findings largely reinforced survey responses, they also served to expand on how CKD clinic providers offered and/or integrated peer support in clinic-based care. Our findings are elaborated across four themes (see Table 2 for illustrative quotes).

**Table 2:** Selected illustrative quotes

**Inconsistent awareness of peer support opportunities**	
*“Maybe once or twice since I’ve worked in the kidney clinic in the last 3 years that I can remember have I heard a nurse say, ‘I told this patient about the peer support program’. They might be doing it. I mean, we all work in the same office. I overhear a lot of their conversations, [but] I haven’t heard them talk about it. I don’t know if the nephrologists talk about it… I don’t think anyone else is talking about it except for me.” –* Social worker 1	
*“I wouldn’t say there’s a lot of awareness. There tends to be around some of the social workers and among those who know the program exists, and we certainly have the pamphlets out there and the information generally available. I wouldn’t say the nursing staff or the dietary staff or the admin staff would be aware of the scope of the program or how to get people connected with it.”* – Social worker 3	
*We don’t know enough about peer support programs and how to train somebody and all these kinds of things…I don’t know much about it; I’ve never attended or anything like that.”* – Social worker 5	
*“I know that they [peer support organization] have the telephone support and an internet program and then I have no idea what goes on in other areas of Canada, but in [city] there was a coffee group started that of course is on hold now because of the pandemic.” –* Nurse 2	
*“I’m not 100% sure of the differences…I think it’s basically the same idea where they you have a person [patient] who is interested in peer support, and then they match you up with someone who [they] think they would be compatible with.” –* Manager 1	
**Logistics of peer support integration in multi-disciplinary care**	
*“We have 1500 patients and there’s one of me, and if I’m the only one who is talking about peer support, I can’t get to everyone… There [isn’t] always a lot of time for me to talk to people. I may be addressing their financial concerns and that is paramount in that moment, rather than being able to look into deeper at those people’s needs and even think about offering peer support.”* – Social worker 1	
*“I think it would be likely that they [patients] use a peer support program if they knew more about it and we were trying to refer everyone to it.”*- Social worker 2	
*“I mean it’s easy to go there [peer support group] and be a guest speaker but doing the recruiting and organizing of space and then mentorship of clients who are willing to be volunteers, that takes a lot of work. The people who are working in these [CKD] programs don’t have enough time to do it off the side of the desk.* – Social worker 7	
*“It’s sort of frustrating because there will be times where you think, ‘I really wish I could take your name and number and give [it] to this person’, or I could [say], ‘You two could get together and start a Facebook group’. But we can’t do that.”* – Nurse 2	
*“I think it [peer support] should be reintroduced because our [CKD] patients change over time…I think it [peer support] basically should be like something that they [clinic staff] check off every time, or it should be introduced periodically, ‘are you sure you now don’t need a bit of peer support.’* – Nephrologist 1	
**Recognition of patient accessibility concerns**	
*“[Peer support program] doesn’t have people who speak languages other than English, which is a huge barrier for people who don’t speak English, and we do have one or two buddies who speak another language besides English.”* – Social worker 1	
*“I haven’t asked, but my thoughts would be [patients] feeling overwhelmed with everything that’s going on. Whether it’s their health or work, just having to do one more thing we are asking them to do. I’ve spoken to a peer support volunteer where she says she hands out her phone number all the time to patients for them to call and she doesn’t even get phone calls.”* – Social worker 8	
*“Sometimes when we are dealing with things geographically in addition to ease with technology. Sometimes you are trying to help those people, but you can’t get it [peer support] to them because them can’t understand or don’t have access to those technologies… We don’t have those teleconference places where people can go into the hospital and use those resources, or they can’t go to the library and use them there.”* – Nurse 1	
*“I promoted that [coffee group] a lot…many didn’t want to drive in from 90 min away for one more thing or were 4 h away so that just won’t work, but really wishing that there was someone [peer] or something [program] that they could make use of.” –* Nurse 2	
*“We have people who don’t want the support and think that everything is okay.”* – Manager 1	
**Integrating support pathways**	
*“I tend to see peer support as part of the team atmosphere. The CKD team’s role is managing the medical needs, psychosocial needs...peer support is more of an adjunct, another layer to the program of being able to answer the questions of what the actual patient experience might look like.”* – Social worker 3	
*“Our patient groups are in silos as well. We [CKD clinic staff] don’t really hear. It’s not often that our peritoneal dialysis folks will be interacting with our hemodialysis folks or they will be come over here [CKD clinic] and interact with our [patients], Even though we are a big department we are kind of on our own at the same time.”* – Social worker 6	
*“I think it would be really useful to liaise with [peer support organization] because they have put so much work and time into a formal program. Then connecting them with social work because they connect with the [organization] for funding support and so they do have a bit of an existing communication process.”* – Nephrologist 2	
*“I think that it would be a great thing to have someone [peer mentor] readily available… Prior to us moving to this location, if we had a patient that was struggling whether or not to get a fistula, there are particular patients that we know that would be more than willing to speak to patients and show them what their fistula looks like. We could easily find out, well, Jane Doe is on dialysis today, I’ll go and ask that person, ‘Can we have a look at your fistula?’ And if so, let’s go have a look.”* – Nurse 3	
*“We do have volunteers here as well. They don’t always come down to clinic, but I guess that is something they probably could start doing. You could do a little visit. It’s more up on our in-centre hemo[dialysis] where they provide a chat or provide tea or coffee… This is a way for them to keep connected with everybody. It would probably help to have them go down to [CKD] clinic and share a few stories, especially on our education days.”* – Manager 2	

#### Inconsistent awareness of peer support opportunities

Awareness of peer support availability varied by role, CKD clinic, and region. Social workers attributed strong awareness to their clinical role, which involved frequent collaboration with organizations offering other support resources. Lack of availability of in-house peer support programs meant that most providers relied on programs offered through external organizations to which interested individuals typically self-referred. Even among individuals aware of these programs, many “didn’t know much about” their format, which impacted their ability to confidently offer them to their patients.

#### Logistics of peer support integration in multi-disciplinary care

Providers reported variable referral practices related to peer support despite a recognized need. Some team members suggested referral should be the primary responsibility of social workers as it naturally fit within their scope of practice, while many social workers indicated that all team members should initiate peer support conversations. Although most agreed that peer support should be offered to all clinic patients given their variable and evolving support needs, they did not have a consistent or systematic way of doing so. Attempts at establishing or offering peer support off the “side of the desk” were complicated by large clinic rosters and competing patient care priorities.

#### Recognition of patient accessibility concerns

Providers acknowledged the importance of addressing accessibility issues to encourage peer support uptake. They expressed the need for culturally and linguistically appropriate options and the importance of connecting patients living in remote locations with local peer mentors. They explained how many patients appeared reluctant to access peer support for reasons including feeling overwhelmed, lacking symptoms, or having to initiate peer interactions. Many suggested embedding peer support into clinic encounters and/or engaging ethnocultural communities in developing and providing peer support as a way of enhancing available options, convenience, and accessibility.

#### Need for integrated support pathways

Providers discussed the importance of connectivity between CKD clinics and organizations offering peer support to encourage access to existing programs and development or integration of new programs within their clinics. Although innovative peer support opportunities were present in some larger kidney programs, such as open houses, buddy programs, and community kitchen initiatives, the fact that most “patient [support] groups are in silos” limited their availability to a broader range of people with CKD. Despite the potential value of integrating peer support within CKD clinics as an “adjunct” to existing support services, providers considered it critical to clarify the scope of services through clear boundaries and expectations.

### Barriers and facilitators to integrating peer support into CKD care

Ranked barriers and facilitators from survey responses and those reported in interviews with supporting quotes are compared in Table 3.

**Table 3:** Barriers and facilitators to offering peer support

**Rank**	**Barriers identified from survey**	**Total rank priority score**	**Top 5 qualitative barriers**
1	Lack of access to program	261	1. Limited awareness of peer support programs (13 participants) Reduced healthcare provider awareness of peer support programs available for individuals with CKD. *“I don’t really know very much about it [peer support], honestly. I just knew that it was supposedly available and initially in some of the package information I had it was available, that they [patients] could reach out and be in touch with others.”* – Nurse 1 2. Challenges of virtual formats (10 participants) Inability to identify and offer peer support to eligible patients due to reduced capacity for in-person encounters. “*To put a damper on it, I really feel like this virus [COVID-19] kind of ruined those [peer support] possibilities.”* – Social worker 6 3. Workload and competing priorities (9 participants) Restricted integration of peer support into CKD care resulting from large clinic rosters with accompanying workloads and competing priorities. *“Some of the clinics are so huge, we may not be able to identify ahead of time which patients may particularly need [peer] support at that time.”* – Nurse 3 4. Perceived patient hesitancy (6 participants) Perceived patient reluctance to initiate peer support conversations or encounters. *“If I put myself in that [patient’s] place… It’s going to take me a while to feel comfortable. I would be very worried about [not] knowing what to say.”* – Nurse 2 5. Lack of time and resources (5 participants) Need for adequate resources to promote, offer, and deliver peer support in CKD clinics. *“You need to find some time in doing that [off] the side of your desk.”* – Social worker 7
2	Lack of awareness of peer support options	255	
3	Workload	246	
4	Lack of resources to provide patient	193	
5	Too much information to provide patients at clinic visits	155	
6	Lack of patient receptivity	111	
7	Limited staff receptivity	37	
8	Feeling uncomfortable talking to patients about peer support	31	
**Rank**	**Facilitators identified from survey**	**Total rank priority score**	**Top 5 qualitative facilitators**
1	Leadership (e.g., local program champion)	294	1. Collaborations between and within organizations (5 participants) Ability to collaborate with other programs and organizations offering peer support. *“I know some other community units were interested [in peer support] and emailed me and asked additional questions. So some other programs picked up on it as well to try to initiate [it].”* - Social worker 7 2. Systematic process for integrating peer support (5 participants) Having consistent processes in place for identifying, discussing, and referring patients to peer support. *“We have two other social workers that will do the referrals as well, and sometimes a nurse might bring it our way and we follow it from there.”* – Social worker 6 3. Staff receptivity (4 participants) Staff engagement in promoting and/or integrating peer support within their clinic. *“I think for the social workers or whoever would be running the groups to keep it consistent and keep going, even if it didn’t work out once to not give up and to keep trying.”* – Social worker 8 4. Patient motivation (4 participants) Patient interest to drive implementation and sustainability of a peer support program *“There are some people that tend to be really keen, they really want to learn from others. They want more information. We thought there would be more interest and more likely people to attend.”* - Social worker 1 5. Strong patient-provider relationships (3 participants) Reliance on trusting relationships between providers and patients for encouraging peer support. *“We can make that connection not only through social work but through nursing with the patients that we are seeing and connect them with peer support.”* – Nurse 3
2	Training and preparation	256	
3	Availability of patient volunteers	254	
4	Assistance with inviting patients to peer support program	204	
5	Management support	195	
6	Funding support	152	
7	Close relationship with external organization	133	
8	Adequate space	127	

*Abbreviation*: *CKD* chronic kidney disease

#### Barriers

Survey respondents emphasized inconsistent awareness of peer support among clinic providers as a main barrier to integrating peer support. Interviews identified additional challenges posed by recent increases in virtual technology use. The latter issue made it difficult to identify patients who might benefit from peer support during clinic encounters and to offer peer support virtually to patients with limited access to technology. Interview and survey participants also identified barriers of workload demands, competing priorities, and lack of adequate time and resources to discuss or offer peer support.

#### Facilitators

Across surveys and interviews, participants underscored having systematic approaches to offering peer support in clinics as a facilitator. This included processes for routine staff education about available programs and clear role definitions among team members as to who discusses peer support with patients. The importance of close relationships with external organizations to foster collaboration in peer support delivery was highlighted more frequently in interviews than surveys. Although leadership and management support were ranked highly as facilitators in the survey, participants did not emphasize this during interviews.

## Discussion

Integrated findings across quantitative and qualitative phases suggest that despite variable awareness of peer support availability, participants appreciated the value of peer support for people with CKD. A lack of systematic processes for introducing peer support within and across CKD clinics meant that many providers were unable to consistently offer it to their patients. Participants expected that attempts at integrating peer support within CKD clinics would be met with challenges, including high workload demands and competing priorities for CKD clinic staff. The importance of addressing accessibility challenges through tailored peer support options was also emphasized across participants. Despite anticipated difficulties, providers offered suggestions for integrating peer support into CKD clinic care with appropriate organizational supports and collaboration.

Participants across roles recognized that provider awareness of peer support was necessary to effectively promote programs to their patients. However, awareness was lower than anticipated and varied by role and region, which may point to variability in how CKD clinics operate and in peer support availability across the country. Although our results differ from other studies reporting higher awareness of peer support among staff in kidney care programs, poor knowledge of peer support practicalities has been identified as a major barrier to uptake of peer support programs among people with varying stages of kidney disease [6, 14, 33]. Promotional strategies that target provider groups with lower awareness, such as CKD clinic nurses in our study, may yield greater impacts on peer support referral rates and uptake [34].

In our study, peer support referral and embeddedness with other clinic support offerings were inconsistent given both limited resources and lack of formalized practices. Participants also described a lack of role clarity related to offering and/or integrating peer support within their clinics. Whereas nephrologists and nurses commonly assumed that social workers were the most appropriate team member to discuss peer support, social workers indicated it should be a shared responsibility. In contrast, respondents from other studies suggested peer support referral fit within the nursing scope of practice [4, 33]. Taken together, findings underscore the critical role of the multi-disciplinary team in enabling patient supports to manage the burden of living with CKD. Regardless of role, providers endorsed the need for systematic processes for offering peer support to patients and their caregivers and indicated that peer support access reflected the high needs of this patient population rather than criticism of the supports available through the care team [7].

Participants emphasized the importance of tailoring peer support delivery to their local context as a way of addressing lower receptivity and access to peer support among CKD patients, yet faced challenges in doing so in the clinic setting. As Canadian CKD clinics operate under regional health authorities and comprise different models of care and resources [19–21, 27, 35], the availability of peer support from external organizations at both national and regional levels may add complexity to how peer support services are accessed through CKD clinics [18]. While national peer support programs can be an effective means for providing peer support to a diverse kidney population, results from this study reinforced the importance of adapting peer support format and delivery to accommodate the geographical, ethnocultural, and socioeconomic complexities of the populations CKD clinics serve [36]. Additionally, the unique requirements of people with non-dialysis-dependent CKD, such as enabling self-management, care transitions, and decision-making related to disease progression, should be considered when offering peer support [2–4]. As such, a concerted approach to peer support delivery that leverages large-scale infrastructure alongside local CKD clinic resources could enhance peer support uptake by attending to patients’ needs and anticipated accessibility challenges.

Wood et al. recently reported on experiences related to the adoption of a national peer support program for patients across various kidney disease contexts, including dialysis, in the United Kingdom (UK) [14]. In this study, providers, peer support users, and peer mentors suggested peer support was well received across settings and that patient and staff optimism and peer support prioritization could enhance program adoption. Identified barriers to peer support uptake included lack of access to information/guidance, reduced staff time, and competing priorities, whereas facilitators included enhanced promotion and use of peer support champions. While we found similar challenges to promotion and uptake, our study focused on the perspectives of providers from multi-disciplinary CKD clinics related to their ability to offer and/or integrate peer support into patient care in a tailored way to those with non-dialysis-dependent CKD.

Findings from our study offer practical considerations for the provision of peer support as part of routine CKD care. CKD clinics may provide an optimal setting for offering or embedding peer support due to their provision of longitudinal care using a multi-disciplinary, team-based approach [19–22]. Our findings suggest that responsive, local peer support options offered in partnership with community organizations could serve the needs of CKD clinics and their patients, and that a single, unified approach to peer support is unlikely to be feasible or effective [36]. Some CKD clinics may have the resources and connections to implement their own in-house peer support program, whereas others may be better suited to external peer support program referral or integration of informal peer support into clinic-based educational programs. The ability to leverage existing peer support structures and learn from other CKD programs could foster sustainable and concerted peer support strategies.

Our study was strengthened by its inclusion of healthcare providers and CKD clinics from across Canada and exploration of complementary aspects of an important care issue for patients with CKD [37]. However, we acknowledge some limitations when interpreting study findings. Although individuals participated from across Canada, we were unable to capture the views of providers from every CKD clinic and from all regions. Moreover, the survey was intended for providers working primarily in non-dialysis-dependent CKD clinics, but due to our snowball sampling approach, few participants provided care exclusively to patients in dialysis and transplant settings. It is also possible that survey and interview responses differed from those who chose not to participate or reflected social desirability bias despite assurances of data de-identification. Regardless, our findings captured a breadth of perspectives across provider roles and regions that are in keeping with other research on how kidney care programs provide care and offer peer support. Lastly, as this study was conducted in the context of CKD care delivery in Canada, findings may have different implications in other settings where funding and program delivery models differ. However, identified preferences, processes, and barriers and facilitators to peer support are likely transferrable to CKD clinicians interested in integrating peer support in other programs.

## Conclusion

In this study we noted variability in how healthcare providers from CKD clinics across Canada promoted and offered peer support opportunities to patients and their caregivers. Our findings suggest that factors such as streamlined referral processes, collaboration between programs, and program adaptation to fit local contexts could encourage peer support awareness and uptake. A clearer picture of how peer support is currently offered to patients with non-dialysis-dependent CKD and providers’ needs related to peer support delivery can inform strategies to optimize its integration into CKD care. Future work to establish and evaluate systematic approaches to peer support in comprehensive CKD care are needed.

## Supplementary Information

**Additional file 1.** Consolidated Criteria for Reporting Qualitative Research (COREQ): completed checklist reporting on qualitative results according to recommended guidelines.

**Additional file 2.** Peer support in CKD survey: questions included in the online survey.

**Additional file 3.** Interview guide: questions used in the semi-structured interviews with a subset of healthcare providers.

**Additional file 4.** Characteristics of in-house peer support: description of integrated peer support programs from respondents indicating peer support delivery within their clinic.

**Additional file 5.** Characteristics of peer support processes: description of clinic processes from respondents indicating peer support awareness.

**Additional file 6.** Likert scale responses: responses to Likert scale questions about perceived need, interest, and impact of peer support.

## Data Availability

We are unable to make our dataset available due to potential identifiability of participating individuals from our qualitative data and survey responses from regional CKD clinic providers. Questions related to primary data can be directed to the corresponding author (MJE).

## Abbreviations

CKD: Chronic Kidney Disease
COREQ: Consolidated Criteria for Reporting Qualitative Research
MMARS: Mixed Methods Article Reporting Standards
UK: United Kingdom
