# Supplementary material for: Healthcare provider perspectives on integrating peer support in non-dialysis-dependent chronic kidney disease care: a mixed methods study
Source: BMC Nephrol. 2022 Apr 18;23:152. doi: 10.1186/s12882-022-02776-w (PMC9014775; doi:10.1186/s12882-022-02776-w)
Supplement: Supplementary file 1 — Additional file 1. Consolidated Criteria for Reporting Qualitative Research (COREQ): completed checklist reporting on qualitative results according to recommended guidelines. [file 12882_2022_2776_MOESM1_ESM.pdf]

Healthcare provider perspectives on integrating peer support in non-dialysis-dependent chronic kidney disease: a mixed methods study

Additional file 1. COREQ 32-item checklist

Item	Description	Reported on Page No.
**Domain 1: Research team and reflexivity**		
1. Interviewer	Shannan Love completed interviews.	7
2. Credentials	Maoliosa Donald, PhD; Meghan Elliott, MD, MSc; Danielle Fox, RN, MN; Tyrone Harrison, MD; Brenda Hemmelgarn, MD, PhD; Shannan Love, MSLP; Nancy Verdin, BScOT	1
3. Occupation	Meghan Elliott, Tyrone Harrison, and Brenda Hemmelgarn are nephrologists and clinician scientists; Maoliosa Donald is an implementation scientist in nephrology; Danielle Fox has a clinical background in nursing and is a PhD candidate in nephrology; Shannan Love is a research coordinator in nephrology; Nancy Verdin has a clinical background in occupational therapy and has lived experience with kidney disease.	1
4. Gender	Meghan Elliott, Brenda Hemmelgarn, Maoliosa Donald, Danielle Fox, Nancy Verdin, and Shannan Love identify as cisgender women; Tyrone Harrison identifies as a cisgender man.	
5. Experience and training	All members of the research team have training in qualitative research methodology and health services research.	7
6. Relationship established	None of the research team members had prior relationships with participants.	7
7. Participant knowledge of the interviewer	The interviewer introduced herself prior to the interview and provided her clinical/research background as appropriate.	7
8. Interviewer characteristics	The interviewer used her first name in all interviews and disclosed her role in the research project. The interviewer shared the aim of the current study which was to better understand healthcare providers' views on peer support provision for people with non-dialysis-dependent CKD. Reflexive notes were taken during all stages of this project, to which the research team referred during the analysis and interpretative phases to refine our findings.	7
**Domain 2. Study design**		
9. Methodological orientation and theory	Qualitative description was the methodology; Conventional content analysis was the analytic approach.	8
10. Sampling	For the online survey, healthcare providers were purposively sampled to provide representation from multi-disciplinary	6

	CKD clinics across Canada. A subset of these providers consented to completing interviews.	
11. Method of approach	A coordinator with the CKD Clinic Network distributed the survey to individuals from CKD clinics across Canada. Interviews were completed with survey respondents who indicated interest at the end of the survey.	6
12. Sample size	18 participants. Sampling continued until the point at which there was a breadth of participants with varied demographic characteristics and perspectives related to peer support delivery, and little new information was emerging from interviews.	7,11
13. Non-participation	Some participants who had initially agreed to an interview were unable to participate. As data saturation was achieved after 18 interviews, no further attempts at recruitment were made.	7
14. Setting of data collection	All interviews were completed by telephone or virtually during the COVID-19 pandemic. Interviews were completed from the research coordinator’s office. Participants completed interviews from their location of choice.	7
15. Presence of non-participants	No non-participants were present for interviews.	N/A
16. Description of sample	See Table 1 for demographic characteristics of all survey respondents and the subset of interview participants.	Table 1
17. Interview guide	See Additional file 3.	7
18. Repeat interviews	Repeat interviews were not undertaken.	N/A
19. Audio/visual recording	All interviews were audio recorded using a handheld recording device.	7
20. Field notes	Field notes were not completed.	N/A
21. Duration	Interviews ranged from 30-60 minutes in duration.	7
22. Data saturation	Data collection and analysis took place concurrently. Data saturation was determined to be the point at which a breadth of participants and perspectives related to peer support delivery had been captured and little or no new information emerged during the analysis.	7
23. Transcripts returned	Interview transcripts were not returned to participants. Participants were invited to contact the research team if they had questions or wanted additional information about the study.	N/A
**Domain 3: Analysis and Findings**		

24. Number of data coders	2	8
25. Description of coding tree	Codes were derived inductively through repeated readings of initial transcripts. Using the first 2-3 transcripts, coders (MJE and SL) independently reviewed transcripts to capture key ideas and concepts from the text. They then met to generate and sort codes into categories based on how they were related to each other. The coders met after completing line-by-line coding of initial transcripts to further discuss analytic thoughts and to refine the coding scheme. Subsequent transcripts were coded by both coders with occasional discussion required primarily related to clarifying code definitions. 22 codes within 4 main categories were derived from the data.	8
26. Derivation of themes	Conventional content analysis was inductive in that codes and themes were derived from the data. Defining and determining relationships between categories helped to develop preliminary themes. Themes were further refined and relationships between themes were explored to provide a descriptive account of participants' view. Exemplars from codes and categories were identified from the data to support themes.	8
27. Software	NVivo 12 (QSR International Pty Ltd)	8
28. Participant checking	The interviewer probed for follow-up on participant comments and summarized participant statements during interviews to ensure complete, accurate information. At the end of each interview, participants were invited to clarify any comments made or to offer additional comments on issues not raised during the session.	N/A
29. Quotations presented	Direct participant quotes are provided throughout the results and in a separate table to support each of the 4 identified themes and barriers and facilitators to integrating peer support into CKD care.	11-13, Tables 2 and 3
30. Data and findings consistent	The data presented are consistent with this study's findings.	11
31. Clarity of major themes	Theme 1 – Inconsistent awareness of peer support opportunities.<br>Theme 2 – Logistics of peer support integration in multi-disciplinary care.<br>Theme 3 – Recognition of patient accessibility concerns.<br>Theme 4 – Need for integrated care pathways.	11-13
32. Clarity of minor themes	No minor themes were identified from the data.	N/A
