# Supplementary material for: Healthcare provider perspectives on integrating peer support in non-dialysis-dependent chronic kidney disease care: a mixed methods study
Source: BMC Nephrol. 2022 Apr 18;23:152. doi: 10.1186/s12882-022-02776-w (PMC9014775; doi:10.1186/s12882-022-02776-w)
Supplement: Supplementary file 2 — Additional file 2. Peer support in CKD survey: questions included in the online survey. [file 12882_2022_2776_MOESM2_ESM.pdf]

Additional file 2. Peer support in CKD survey questions

PURPOSE: Peer support refers to programs and services that connect people with chronic kidney disease (CKD) with others who have lived experience of CKD. Peer support can provide different perspectives, emotional support, and can help to normalize the CKD experience. We would like to better understand how multi-disciplinary team members integrate peer support into CKD care. We will ask you about any peer support programs that are offered through your CKD clinic.

HOW YOU WILL BENEFIT: The information collected will be used to help inform integration of peer support into comprehensive CKD care provided in CKD clinics.

HOW YOU CAN HELP: Please complete the survey - it will take approximately **10 minutes**. The questions will ask about your awareness of peer support programs, your views on the value of peer support for people with CKD, and how CKD clinics are offering or integrating peer support into their clinic.

When you are answering, please think specifically about the care of **people with non-dialysis CKD** followed in **multi-disciplinary CKD clinics**.

PARTICIPATION: While your participation is voluntary, we would appreciate your input to ensure that we have a full picture of peer support in CKD care across Canada. Survey responses will be kept confidential, and results will be presented in aggregate form. **Completion of the survey indicates your consent to participate**.

Thank you for your participation.

We want to learn a little more about you and the clinic where you work.

Q1 Name of CKD clinic:

Q2 Size of city/town where clinic is located:

- o Less than 100,000
- o 100,000 - 499,000
- o 500,000 - 1,000,000
- o More than 1,000,000

Q3 City/town clinic is located:

Q4 Type of patients seen in your clinic: (select all that apply)

- ☐ Stages 1-3 CKD (eGFR $\geq$30 mL/min)
- ☐ Stages 4 and 5 non-dialysis CKD (eGFR <30 mL/min)
- ☐ Hemodialysis
- ☐ Peritoneal dialysis
- ☐ Transplant
- ☐ Other (please specify):
- ☐ Unsure

Q5 Approximate number of patients with non-dialysis CKD (i.e., eGFR <30 mL/min) followed in your clinic:

- Under 500
- 500 - 1000
- Over 1000
- Unsure

Q6 Your role in the clinic: (select more than one if applicable)

- ☐ Nephrologist
- ☐ Nurse
- ☐ Social worker
- ☐ Spiritual care practitioner
- ☐ Pharmacist
- ☐ Dietitian
- ☐ Kinesiologist
- ☐ Physical therapist
- ☐ Occupational therapist
- ☐ Clinic or unit manager
- ☐ Support staff
- ☐ Other (please specify):

Q7 Type of nurse:

- ☐ Nurse practitioner
- ☐ Registered nurse
- ☐ Licensed practical nurse
- ☐ Health care aide / Nursing assistant

Q8 Who is part of your CKD clinic care team: (select all that apply)

- ☐ Nephrologist
- ☐ Nurse Practitioner
- ☐ Registered nurse
- ☐ Licensed practical nurse
- ☐ Health care aide / Nursing assistant
- ☐ Social worker
- ☐ Spiritual care practitioner
- ☐ Pharmacist
- ☐ Dietitian
- ☐ Kinesiologist
- ☐ Physical therapist
- ☐ Occupational therapist
- ☐ Support staff
- ☐ Other (please specify):

Q9 Length of time in current position:

- Less than 1 year
- 1 - 5 years
- 6 - 10 years
- More than 10 years

We would like to know more about your awareness and the availability of peer support.

A CKD peer support program is a program that provides additional support to patients with CKD and their caregivers by bringing them together with trained peers who have lived experience with kidney disease. Examples of peer support programs are in-person groups, telephone calls, and facilitated forums or social media groups (e.g., Facebook).

Q10 Are you **aware of any peer support programs** available for people with CKD (i.e., offered through your health region, CKD program, or external organization)?

- Yes - If yes, please list:
- No

Q11 Do you refer your CKD patients to any of these peer support programs?

- ☐ Yes - If yes, which ones?
- ☐ No

Q12 How did you learn about peer support programs for people with CKD? (select all that apply)

- ☐ During staff orientation
- ☐ Informally from other clinic staff
- ☐ Patients/caregivers inform staff
- ☐ In-services or staff education sessions
- ☐ Individuals or organizations outside the clinic
- ☐ Emails, newsletters
- ☐ Other (please specify):
- ☐ Unsure

Q13 Are any of these peer support programs delivered by your CKD clinic (e.g., support groups, one-on-one sessions)?

- Yes
- No

The following series of questions refers to **peer support programs delivered by your clinic**. Please complete these questions **once for each program** that your clinic delivers. If you offer more than one program, you will be prompted to complete these questions again.

Q14 Name of program:

Q15 Who is the program intended for? (select all that apply)

- ☐ Patients with CKD
- ☐ Caregivers of patients with CKD
- ☐ Other (please specify):
- ☐ Unsure

Q16 Who facilitates the peer support program? (select all that apply)

- ☐ Healthcare provider
- ☐ Patient volunteer
- ☐ Partner organization (e.g., Kidney Foundation of Canada)
- ☐ Unfacilitated
- ☐ Other (please specify):
- ☐ Unsure

Q17 Type of healthcare provider:

- ☐ Physician
- ☐ Nurse
- ☐ Social worker
- ☐ Spiritual care practitioner
- ☐ Other (please specify):
- ☐ Unsure

Q18 What is the format of this program? (select all that apply)

- ☐ One on one
- ☐ Small group (less than 10 participants)
- ☐ Large group (more than 10 participants)
- ☐ Other (please specify):
- ☐ Unsure

Q19 How is the program offered? (select all that apply)

- ☐ Single event or session
- ☐ Recurring session or program
- ☐ Initial event with option for ongoing follow-up
- ☐ Other (please specify):
- ☐ Unsure

Q20 Type of recurring session or program: (select all that apply)

- ☐ On-going sessions
- ☐ Set number of sessions
- ☐ Other (please specify):
- ☐ Unsure

Q21 The program is offered as a:

- ▢ Registered program (i.e., consistent participants)
- ▢ Drop in (i.e., changing participants or as needed)
- ▢ Other (please specify):
- ▢ Unsure

Q22 What is the mode of delivery? (select all that apply)

- ▢ In person
- ▢ By telephone
- ▢ By virtual platform (e.g., Skype, Zoom)
- ▢ Online blog or social media platform (e.g., Facebook group)
- ▢ Other (please specify):
- ▢ Unsure

Q23 Additional features of the peer support program: (select all that apply)

- ▢ Follows an existing program or curriculum (e.g., Heart Math, coping skills development) Please specify:
- ▢ Includes an educational component
- ▢ Includes opportunities for informal support (e.g., social outings, coffee meetings)
- ▢ Other (please specify):
- ▢ Unsure

Q24 Do you provide any other programs through your clinic?

- o Yes
- o No

We would now like to know **how patients and caregivers learn about peer support opportunities** (both in-clinic and external programs).

Q25 How do patients who come to your clinic and their caregivers learn about peer support opportunities? (select all that apply)

- ▢ Directly from CKD clinic staff
- ▢ Advertising in newsletters
- ▢ Posters in waiting room
- ▢ Brochures, bookmarks, other handouts available to patients during clinic visits
- ▢ During new patient orientations
- ▢ During group education sessions
- ▢ Peer support volunteers talking to patients in clinic
- ▢ Our clinic does not share peer support resources
- ▢ Other (please specify):
- ▢ Unsure

Q26 When is peer support discussed with CKD patients and their caregivers? (select all that apply)

- ▢ It isn't routinely discussed in the CKD clinic program
- ▢ Upon entry to CKD clinic program only
- ▢ Upon entry to CKD clinic program, then reintroduced periodically as needed
- ▢ When patient or caregiver appears to need additional support
- ▢ When patient is facing significant decisions related to treatment or life events
- ▢ Other (please specify):
- ▢ Unsure

Q27 In your clinic, who is offered peer support? (select all that apply)

- ▢ All CKD patients who attend the clinic
- ▢ CKD patients with higher support needs (as determined by clinic staff)
- ▢ All caregivers of CKD patients who attend the clinic
- ▢ Caregivers of CKD patients with higher support needs
- ▢ Other (please specify):
- ▢ Our clinic does not offer peer support to patients or caregivers
- ▢ Unsure

Q28 What is the process in your clinic for referring patients and their caregivers to peer support program(s)? (select all that apply)

- ▢ Staff member refers patient to program
- ▢ Patient self-refers to program
- ▢ Peer support volunteer talks to patient in clinic, then refers to program
- ▢ Other (please specify):
- ▢ Our clinic does not refer patients or caregivers to peer support
- ▢ Unsure

Q29 What type of support need could trigger a referral for peer support? (select all that apply)

- ▢ Informational support (e.g., information on CKD, treatment options)
- ▢ Emotional support (e.g., help with coping, mental health issues)
- ▢ Appraisal support (e.g., talking to someone with lived experience for reassurance and to normalize the experience)
- ▢ Instrumental support (e.g., help with meal preparation, attending appointments)
- ▢ Other (please specify):
- ▢ Unsure

Q30 Are there any **informal opportunities** for patients to connect with other patients through your clinic? (e.g., education sessions, introducing patients to other patients outside a peer support program, social outings, waiting room)

- Yes - If yes, please describe:
- No
- Unsure

Q31 What do you think are the **barriers or challenges to offering peer support** opportunities to CKD patients in your clinic? (select all that apply)

- ▢ Lack of awareness of peer support options
- ▢ Feeling uncomfortable talking to patients about peer support
- ▢ Lack of access to program (e.g., space, distance, transportation, parking)
- ▢ Workload (e.g., competing priorities)
- ▢ Lack of patient receptivity
- ▢ Limited staff receptivity
- ▢ Lack of resources to provide patients
- ▢ Too much information to provide to patients at clinic visits
- ▢ Other (please specify):
- ▢ Other (please specify if additional barrier):
- ▢ Unsure
- ▢ No barriers or challenges

Q32 Rank your selections from biggest barrier/challenge to smallest by dragging and dropping the options. Place **the biggest barrier at the top/#1**.
______ Lack of awareness of peer support options
______ Feeling uncomfortable talking to patients about peer support
______ Lack of access to program (e.g., space, distance, transportation, parking)
______ Workload (e.g., competing priorities)
______ Lack of patient receptivity
______ Limited staff receptivity
______ Lack of resources to provide patients
______ Too much information to provide to patients at clinic visits
______ Other (please specify):
______ Other (please specify if additional barrier):
______ Unsure
______ No barriers or challenges

Q33 Which of the following factors would help to **encourage peer support** within your clinic? (select all that apply)

- ▢ Leadership (i.e., someone to "champion" the program)
- ▢ Training and preparation
- ▢ Management support
- ▢ Close relationship with external organizations
- ▢ Assistance with inviting patients to peer support program (e.g., on-site patient volunteers)
- ▢ Funding support
- ▢ Adequate space
- ▢ Availability of patient volunteers
- ▢ Other (please specify):
- ▢ Other (please specify additional facilitator):
- ▢ Unsure
- ▢ No facilitators

Q34 Rank your selections from the most to least helpful by dragging and dropping the options. Place the **most helpful option at the top/#1**.

______ Leadership (i.e., someone to "champion" the program)
______ Training and preparation
______ Management support
______ Close relationship with external organizations
______ Assistance with inviting patients to peer support program (e.g., on-site patient volunteers)
______ Funding support
______ Adequate space
______ Availability of patient volunteers
______ Other (please specify):
______ Other (please specify additional facilitator):
______ Unsure
______ No facilitators

Lastly, we would like to know how you view the need and value of peer support for patients and caregivers in your clinic.

Q35 How do you view the **need** for peer support among CKD patients and their caregivers in your clinic?

Low or no **need**					Medium **need**				High **need**		
0	1	2	3	4	5	6	7	8	9	10	N/A

Q36 How would you rate the **interest** of staff within your clinic to offer peer support resources or programs as part of CKD care?

Low or no **interest**				Medium **interest**				High **interest**			
0	1	2	3	4	5	6	7	8	9	10	N/A

Q37 For patients or caregivers who have accessed peer support, how do you think it has **impacted** their care?

Low or no **impact**				Medium **impact**				High **impact**			
0	1	2	3	4	5	6	7	8	9	10	N/A

Q38 How would you rate the **interest** of CKD patients who attend the CKD clinic and their caregivers in receiving peer support?

Low or no **interest**				Medium **interest**				High **interest**			
0	1	2	3	4	5	6	7	8	9	10	N/A

Q39 Thank you for your participation. We appreciate and value your input regarding peer support in CKD care. If you have any additional comments please add them below:

Q40 Can we contact you to discuss your views on CKD peer support in more detail? We will be completing a short virtual or phone interview that will take approximately 20 minutes.

- Yes
- No

Name:
Email:
Phone number:
