# Supplementary material for: Healthcare provider perspectives on integrating peer support in non-dialysis-dependent chronic kidney disease care: a mixed methods study
Source: BMC Nephrol. 2022 Apr 18;23:152. doi: 10.1186/s12882-022-02776-w (PMC9014775; doi:10.1186/s12882-022-02776-w)
Supplement: Supplementary file 3 — Additional file 3. Interview guide: questions used in the semi-structured interviews with a subset of healthcare providers. [file 12882_2022_2776_MOESM3_ESM.pdf]

Additional file 3. Interview guide

1. Tell me about any peer support program(s) that you are aware of or that your clinic is involved with for people with chronic kidney disease (CKD).

   Probes:

   - How did you find out about the program(s)?
   - Tell me about the program (e.g., format, location, who attends/facilitates)
   - How does your clinic incorporate peer support into CKD care, if at all?
   - How do you/your clinic engage with other organizations that offer peer support?

2. How do clinic staff learn about peer support opportunities for patients with chronic kidney disease?

   Probes:

   - What information or training did you receive about the program(s)?
   - How does peer support align with the goals/objectives of your program or clinic?

3. How do patients and family members in your clinic learn about peer support opportunities?

   Probes:

   - What promotional/recruitment materials do you make available? What is the referral process?
   - How is this process working? Why do you think this process has been successful (or not)?

4. What role do you think peer support could play in the care of your clinic's CKD patients?

   Probes:

   - What types of support do you find CKD patients and their families are requesting? How do you think peer support could meet those needs?
   - What are the limitations to the support clinicians can offer in the clinic?
   - How do you think support needs have changed during the pandemic?

5. What do you think the ideal peer support program would look like for patients with advanced, non-dialysis CKD?

   Probes:

   - What do you think are the barriers to offering or integrating peer support in your clinic? What would help?
   - What are the barriers for patients to access peer support?
   - What suggestions do you have for integrating peer support into your CKD program?
