# Supplementary material for: Healthcare provider perspectives on integrating peer support in non-dialysis-dependent chronic kidney disease care: a mixed methods study
Source: BMC Nephrol. 2022 Apr 18;23:152. doi: 10.1186/s12882-022-02776-w (PMC9014775; doi:10.1186/s12882-022-02776-w)
Supplement: Supplementary file 4 — Additional file 4. Characteristics of in-house peer support: description of integrated peer support programs from respondents indicating peer support delivery within their clinic. [file 12882_2022_2776_MOESM4_ESM.pdf]

Additional file 4. Characteristics of in-house peer support programs (from n=14 survey respondents indicating peer support availability through a clinic program)

Characteristic	Number[a]
Who program is intended for:	
Patients with CKD	10
Caregivers of patients with CKD	8
Unsure	1
Who facilitates the peer support program:	
Healthcare provider	6
Patient volunteer	6
Partner organization	6
Unfacilitated	1
Format of peer support program:	
One on one	6
Small group (< 10 participants)	6
Large group (> 10 participants)	2
How the program is offered:	
Single session or event	4
Recurring session or program	5
Initial event with option for follow-up	2
Other (e.g., on individual basis, on hold due to pandemic)	3
Registered program (i.e., consistent participants)	4
Drop in (i.e., changing participants as needed)	4
Other (e.g., as part of education session)	5
Mode of delivery:	
In-person	12
By telephone	7
By virtual platform (e.g., Skype, Zoom)	1
Other	3
Additional features of peer support program:	
Educational component	3
Opportunities for informal peer support	5
Unsure	4

Note: These questions were presented only to respondents who reported offering in-house peer support in the survey.
[a]Some respondents did not respond to every question offered or selected more than one response, therefore each section does not necessarily total n=14.
Abbreviation: CKD, chronic kidney disease.
