# Supplementary material for: Healthcare provider perspectives on integrating peer support in non-dialysis-dependent chronic kidney disease care: a mixed methods study
Source: BMC Nephrol. 2022 Apr 18;23:152. doi: 10.1186/s12882-022-02776-w (PMC9014775; doi:10.1186/s12882-022-02776-w)
Supplement: Supplementary file 5 — Additional file 5. Characteristics of peer support processes: description of clinic processes from respondents indicating peer support awareness. [file 12882_2022_2776_MOESM5_ESM.pdf]

Additional file 5. Characteristics of peer support processes (from n=75 survey respondents indicating peer support awareness)

Characteristic	Number (%)[a]
How healthcare providers learn about peer support programs	
Individuals or organizations outside the clinic	40 (53.3)
Informally from other clinic staff	39 (52.0)
In-services or staff education sessions	28 (37.3)
During staff orientation	21 (28.0)
Emails, newsletters	21 (28.0)
Patients and caregivers inform staff	9 (12.0)
Other (e.g., internet searches, have always known)	6 (8.0)
How patients and caregivers learn about peer support opportunities	
Directly from CKD staff	62 (82.7)
Brochures and handouts	44 (58.7)
Posters in waiting room	30 (40.0)
New patient orientation	25 (33.3)
Group education sessions	15 (20.0)
Advertising in newsletters	14 (18.7)
Peer support volunteer approaching patients in clinic	5 (6.7)
Other (e.g., websites)	2 (2.7)
Clinic does not share peer support resources	1 (1.3)
Unsure	4 (5.3)
Who is offered peer support by clinic staff	
CKD patients with higher support needs	27 (36.0)
All CKD patients who attend clinic	24 (32.0)
All caregivers of CKD patients who attend clinic	14 (18.7)
Caregivers of CKD patients with higher support needs	12 (16.0)
Other (e.g., patient/caregiver directly requests, not routinely offered)	6 (8.0)
Clinic does not offer peer support to patients or caregivers	3 (4.0)
Unsure	12 (16.0)
When peer support is discussed with patients and caregivers	
When patient or caregiver appears to need additional support	38 (50.7)
When patient is facing significant decisions	27 (36.0)
Not routinely discussed	23 (30.7)
Upon entry to CKD clinic program, then reintroduced periodically as needed	21 (28.0)
Other (e.g., when patient asks, embedded into education sessions)	5 (6.7)
Upon entry to CKD clinic program only	3 (4.0)
Unsure	6 (8.0)
Process for referring patients and their caregivers to peer support programs	
Patient/caregiver self-refers to program	49 (65.3)
Staff member refers patient/caregiver to program	27 (36.0)
Other (e.g., no specific process, patient directly linked to peer volunteer in clinic)	6 (8.0)
Clinic does not refer patients/caregivers to peer support	3 (4.0)
Peer support volunteer talks to patient/caregiver in clinic, then refers to program	2 (2.7)
Unsure	7 (9.3)

Type of support that could trigger referral to peer support	
Emotional support	53 (70.7)
Appraisal support	50 (66.7)
Informational support	48 (64.0)
Instrumental support	12 (16.0)
Other (e.g., support with transitions to other modalities)	1 (1.3)
Unsure	2 (2.7)

Note: These questions were presented only to respondents who reported being aware of peer support programs.
[a]Many respondents chose more than one response for each question, therefore each section does not necessarily total n=75.
Abbreviation: CKD, chronic kidney disease.

Healthcare provider perspectives on integrating peer support in non-dialysis-dependent chronic kidney disease: a mixed methods study
