# Supplementary material for: Healthcare provider perspectives on integrating peer support in non-dialysis-dependent chronic kidney disease care: a mixed methods study
Source: BMC Nephrol. 2022 Apr 18;23:152. doi: 10.1186/s12882-022-02776-w (PMC9014775; doi:10.1186/s12882-022-02776-w)
Supplement: Supplementary file 6 — Additional file 6. Likert scale responses: responses to Likert scale questions about perceived need, interest, and impact of peer support. [file 12882_2022_2776_MOESM6_ESM.pdf]

Additional file 6. Likert scale responses (from n=113 survey respondents)

Survey question	Number of respondents	Median score[a] (IQR)
How do you view the need for peer support among CKD patients and their caregivers in your clinic?	94	7.5 (6,9)
How would you rate the interest of staff within your clinic to offer peer support resources or programs as part of CKD care?	72	7 (6,9)
For patients or caregivers who have accessed peer support, how do you think it has impacted their care?	79	6 (5,7)
How would you rate the interest of CKD patients who attend the CKD clinic and their caregivers in receiving peer support?	94	6 (4,8)

[a]Likert scale options ranged from 0 (low) to 10 (high).
